# Effect of financial stress on self-rereported health and quality of life among older adults in five developing countries: a cross sectional analysis of WHO-SAGE survey

**DOI:** 10.1186/s12877-020-01687-5

**Published:** 2020-08-12

**Authors:** Rui Huang, Bishwajit Ghose, Shangfeng Tang

**Affiliations:** 1 School of Pharmacy, Tongji Medical College, Huazhong University of Science and Technology, Wuhan, 430030 China; 2 School of Medicine and Health Management, Tongji Medical College, Huazhong University of Science and Technology, Wuhan, China

**Keywords:** Financial stress, Subjective health, Quality of life, Older adults, WHO-SAGE study

## Abstract

**Abstract:**

In developing countries, older adults (65 years of age and above) share an increased vulnerability to catastrophic health expenditures and financial stress which can have significant bearing on their health and well-being. Currently, research evidence on how financial stress correlates with health and well-being among older adults in the developing countries is limited. Therefore, in this study, we aimed to assess the relationship between financial stress and subjective 1) health, 2) depression, 3) quality of life, and 4) life satisfaction among older adults in five developing countries.

**Methods:**

Data used in this study were cross-sectional which were collected from the first wave of Study on Global AGEing and Health (SAGE) survey of World Health Organization. Sample population were 12,299 community dwelling men and women in China (*n* = 4548), Ghana (*n* = 1968), India (*n* = 2441), South Africa (*n* = 1924), and Russia (*n* = 1418). Using generalized linear models with logit links, we assessed the correlation between self-reported financial stress and income inequality with the four outcome measures by adjusting for various sociodemographic factors.

**Results:**

Overall, the prevalence of good self-reported health, quality of life and positive life-satisfaction was 47.11, 79.25 and 44.40% respectively, while 20.13% of the participants reported having depression during past 12 months. Only about a fifth (18.67%) of the participants reported having enough money to meet daily their necessities completely, while more than quarter (28.45%) were in the lowest income quintile. With a few exceptions, the odds of reporting good self-reported health, quality of life, and life satisfaction were generally lower among those with varying degrees of financial stress, and larger among those in the higher income quintiles. Conversely, the likelihood of self-reported depression was significantly higher among those with any level of financial stress, and lower among those in the higher income quintiles.

**Conclusion:**

This study concludes that both subjectively and objectively measured financial stress are inversely associated with good self-reported health, quality of life, life satisfaction, and positively associated with self-reported depression among older adults.

## Background

Last few decades in the developing world have been marked with success stories in population health surrounding improved nutrition and child survival, falling female fertility rates, better living standard and increasing longevity [1–4]. Countries differ significantly in terms of their pace and magnitude of progress in these areas, and experience varying degrees of population aging, also known as the Third Demographic Transition [5]. The concern regarding population aging and the associated health and socioeconomic consequences are relatively higher in the developed economies [6, 7], who also account for bulk of the research and investment on this subject. The emerging economies, on the other hand, have a predominantly younger age structure and have smaller segment of the middle aged (typically between 45 and 64 years) and older adults (> 64 years).

Despite this conducive demographic profile for labour market and healthcare systems, the repercussions of aging on population health and development efforts are no less challenging for the developing countries [8]. In fact, the socioeconomic impacts of population aging in the developing countries is more pronounced owing to inadequate resource and logistical capacity and healthcare infrastructure to provide quality care for the elderly [9]. These challenges are mutually disadvantageous and cyclical in nature in the sense that lack of national capacity can limit the provision of much needed financial insecurity and social and health for the elderly, which in turn can affect the economy through fueling poverty, diseases, and social distress [10].

Elderly people, in comparison with other working-age population, bear significantly higher risk of financial stress due to diminishing physical and mental ability and vice versa [11, 12]. In the context of resource-constraint settings, the situation of elderly poverty is more critical due to the absence of effective social safety nets, lower coverage of health insurance, and the necessary social, transport and recreational infrastructure to meet the special needs of the old age [13, 14]. And unfortunately, lower research capacity and lack of appreciation of the issue deter the development of necessary policies and interventions to promote health and well-being of the population. To this regard, we conducted the present study using data from the Study on global AGEing and adult health (SAGE) conducted by World Health Organization between 2007 and 2010 covering the following six: China, India, Ghana, Russia and South Africa. The SAGE survey is available through GATEWAY TO GLOBAL AGING DATA and is one the group of surveys dedicated to collecting quality data on population aging across the continents. The analysis consisted of assessing the effect of financial stress on self-rated health and quality of life among older people in developing countries.

## Methods

### Data source

Data used in this study were collected from the first wave of Study on Global AGEing and Health (SAGE) survey of World Health Organization. SAGE is a longitudinal and nationally representative survey that included community dwelling population aged 50 years in China, Ghana, India, Mexico, Russia and South Africa [15]. However, at the time of conducting the analysis, data from only the first wave of the surveys were available in the public domain. Therefore, the data used in this study are essentially cross-sectional in nature. Mexico was not included in the analysis sue to several missing variables that were the main focus of the study. The sampling was arranged so that some households will interview all residents aged 50 years and older, while other households would select a person aged 18 to 49 years old. The present study only included population 65 years and above. Sample population for the surveys in each country were selected using multi-stage cluster design. WHO-SAGE data sets are in the public domain and the details of survey methods are published elsewhere [16–19].

### Measures

The outcome measures included self-reported 1) health (SRH), 2) depression, 3) quality of life, and 4) life satisfaction.

Self-reported health was assessed by the question: How do you rate your health today? [20–22]” with the answers ranging from Very Good, Good, Moderate, Bad to Very Bad. For analytical purposes, the answers were collapsed into two categories: good SRH (very good & good), and not-good (Moderate & Bad & Very Bad). Self-reported depression was assessed by the question: “During the last 12 months, have you had a period lasting several days when you felt sad, empty or depressed? [23–25]” The answers were kept as a binary response: “Yes” and “No”. Quality of life was also measured subjectively by the question: “How would you rate your overall quality of life?” with the answers being: Very Good, Good, Moderate, Bad, Very Bad. Similar to SRH, quality of life was recoded as: Good (very good & good), and not-good (Moderate & Bad & Very Bad). Life satisfaction was assessed by the question: “Taking all things together, how satisfied are you with your life as a whole these days?’ The answers were: Very Satisfied; Satisfied; Neither Satisfied; Nor Dissatisfied; Dissatisfied; Very Dissatisfied, and was recoded as: Satisfied (Very satisfied & Satisfied) and Not-satisfied (Nor Dissatisfied & Dissatisfied & Very Dissatisfied) [26–29].

The main explanatory variables were financial situation. The surveys included both subjective and objectives measure of financial stress for all individuals. For this study, subjective financial stress was assessed by the following question: Money sufficient for daily living? To which participants could answer: Completely; Moderately; A Little; Not at All [30–32]. The survey also collected information on income status which was used to rank the participants into quintiles: Q1 (lowest income quintile) to Q5 (highest income quintile) [33–36].

The sociodemographic covariates (potentially confounding factors) were selected based on a review of the past studies on similar themes. The review included studies conducted both on elderly and other age groups. The following were found to be the recurring items in most studies: Age (65–69/70–74/75–79/80–84/85+); Sex (Male/Female); Currently married (No/Yes); Education (None/Primary/Secondary/Higher); Has employment (Yes/No); Residence (Urban/Rural); Smoking (No/Yes); Alcohol user (No/Yes); Takes physical exercise (No/Yes); Has any NCDs (No/Yes). For NCDs, the following conditions were included: asthma, angina, back pain, cataract, diabetes, depression, edentulism, hypertension, obesity. Similar to the outcome variables, these items were also self-reported and coded binarily as Yes (has any conditions) and No (no condition) [15–17, 23, 37–40].

### Data analysis

Statistical analysis was carried out using Stata 14 for Windows. At first, the dataset was checked to ensure the study population were correctly defined (aged 65 years and above). Participants who didn’t have data on the outcome variables were excluded from the analysis. All the variables were screened for missing values and outliers. Initial bivariate tests were conducted to check whether all the explanatory variables were significantly associated with at least one of the outcome variables. The first step of the analysis included descriptive analysis to show the distribution of the each of the outcome measures along the explanatory variables. Following that, a series of multiple logistic regression models (generalised linear models with logit link) were run to test the association between the outcome and explanatory variables. Three different models were performed for each of the four outcomes: 1) including subjective financial stress only, 2) including income quintile only, 3) including both subjective financial stress and income quintile. Strength of these associations were presented as odds ratios (OR) with 95% confidence intervals (95% CI). The value of *p* < 0.05 was considered as statistically significant for all analyses.

### Ethics statement

The study was based on publicly available anonymised data; therefore, no IRB approval was necessary. SAGE surveys were approved by WHO, and informed written consent was obtained from all participants.

## Results

Table 1 shows the sociodemographic profile of the sample population for individual countries. Total sample comprised of 41% men and 59% women, and a greater proportion of them were aged 70–74 years (34.61%). The prevalence of good self-reported health and quality of life were 47.11, and 79.25% respectively, while 20.13% of the participants reported having depression during past 12 months. Less than half (44.40%) of the participants reported being satisfied with life. 14.18% of the participants were in the highest income quintile, however, 18.67% of the reported having enough money to meet daily necessities completely.

**Table 1:** Sociodemographic characteristics of the sample population

	***N*** = 12,299		China (*n* = 4548)	Ghana (*n* = 1968)	India (*n* = 2441)	S. Africa (*n* = 1924)	Russia (*n* = 1418)	***X***^**2**^ ***p***-value
**Age groups**								
65–69	4010	32.60	32.32	14.04	26.31	13.77	13.57	< 0.001
70–74	4257	34.61	46.37	14.63	16.8	13.27	8.93	
75–79	2446	19.89	43.30	14.60	14.02	17.70	10.38	
79+	1586	12.90	13.87	26.80	20.68	23.64	15.01	
**Sex**								
Male	5050	41.06	31.53	18.99	26.38	12.16	10.93	< 0.001
Female	7249	58.94	40.78	13.92	15.3	18.09	11.92	
**Currently married**								
No	5962	48.48	32.52	18.13	16.2	19.04	14.11	< 0.001
Yes	6337	51.52	41.19	14.00	23.28	12.47	9.07	
**Education**								
None	5893	47.92	30.29	25.93	28.83	1.80	13.15	< 0.001
Primary	2024	16.46	47.68	7.36	15.46	14.43	15.07	
Secondary	1797	14.61	50.31	2.56	9.74	28.27	9.13	
Higher	2585	21.01	34.57	9.49	9.83	39.45	6.66	
**Has employment**								
Yes	3100	29.53	36.26	33.65	20.13	5.03	4.94	< 0.001
No	7399	70.47	34.77	12.00	15.38	23.65	14.19	
**Residency**								
Urban	7646	62.18	49.82	10.02	8.21	19.91	12.05	< 0.001
Rural	4653	37.82	15.91	25.84	38.98	8.66	10.6	
**Smoking**								
No	6794	55.21	27.23	21.19	16.04	21.72	13.83	< 0.001
Yes	5515	44.79	49.00	9.60	24.54	8.17	8.69	
**Alcohol user**								
No	7109	57.79	35.5	12.31	29.31	8.20	14.67	< 0.001
Yes	5192	42.21	39.0	21.05	6.88	25.85	7.22	
**Takes physical exercise**								
No	4307	35.04	29.07	13.7	22.31	14.33	20.59	< 0.001
Yes	7985	64.96	41.18	17.26	18.53	16.38	6.65	
**Has any NCDs**								
No	3259	26.55	39.37	22.61	19.24	4.60	14.18	< 0.001
Yes	9014	73.45	35.92	13.66	20.12	19.69	10.61	
**Money meets daily need**								
Completely	2283	18.67	37.23	4.51	19.89	31.98	6.40	< 0.001
Moderately	3578	29.26	35.66	10.87	28.31	16.35	8.80	
A Little	4036	33.00	41.85	20.22	16.58	7.95	13.40	
Not at All	2332	19.08	31.42	27.52	12.99	11.62	16.46	
**Income Quantile**								
Q1	3490	28.45	56.33	12.26	11.89	12.32	7.19	< 0.001
Q2	1986	16.18	18.34	21.31	23.68	22.97	13.70	
Q3	3290	26.82	52.07	12.43	13.95	13.31	8.24	
Q4	1764	14.38	16.61	21.03	27.04	18.14	17.18	
Q5	1739	14.18	12.13	19.26	35.02	15.93	17.65	
**Self-reported health**								
Good	5795	47.11	40.53	15.79	19.36	8.75	15.57	< 0.001
Not-Good	6504	52.89	33.81	16.19	20.27	21.80	7.93	
**Self-reported depression**								
No	9823	79.87	37.78	16.65	18.77	13.52	13.27	< 0.001
Yes	2476	20.13	33.76	13.41	24.11	24.11	4.60	
**Perceived quality of life**								
Good	9747	79.25	40.75	15.47	17.58	16.59	9.61	< 0.001
Not-good	2552	20.75	22.57	18.03	28.49	12.07	18.85	
**Life satisfaction**								
Satisfactory	6124	49.80	44.40	16.19	15.82	16.37	7.22	< 0.001
Not-satisfactory	6175	50.20	29.62	15.81	23.84	14.93	15.81	

**Fig. 1:**
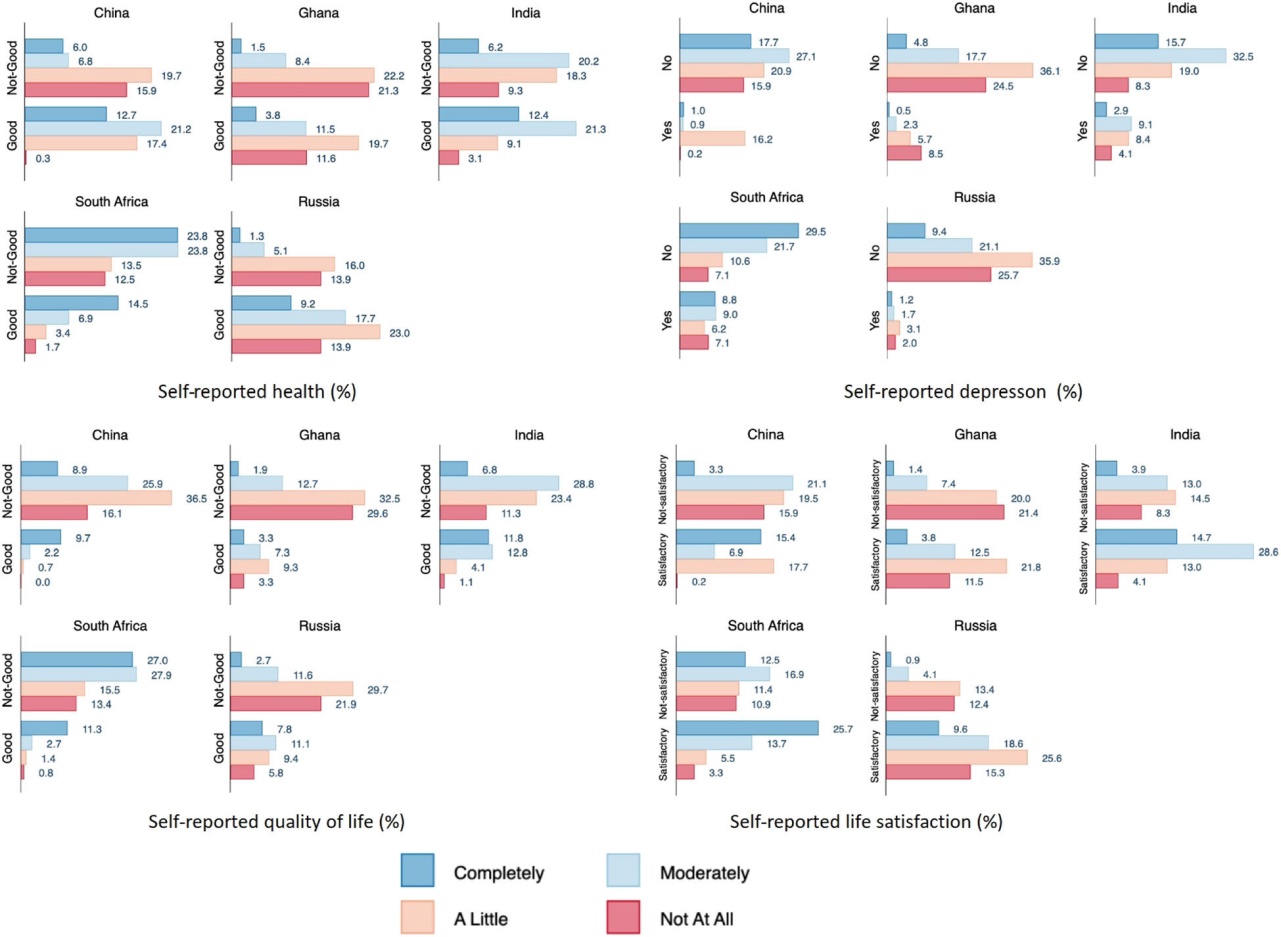
Prevalence of self-reported health, depression, quality of life and life satisfaction by subjective financial stress (%)

Figure 1 shows that the percentage of participants who reported good health and quality of life were relative higher among those who reported meeting daily financial needs ‘Completely’ compared with those who mentioned having varying degrees of difficulty. Conversely, the percentage of self-reported depression was relatively higher among those who could meet daily financial needs ‘Completely’ compared with other. For life-satisfaction, having enough money to meet daily needs ‘Completely’ also had relatively higher percentage of reporting satisfaction with life in all five countries

**Fig. 2:**
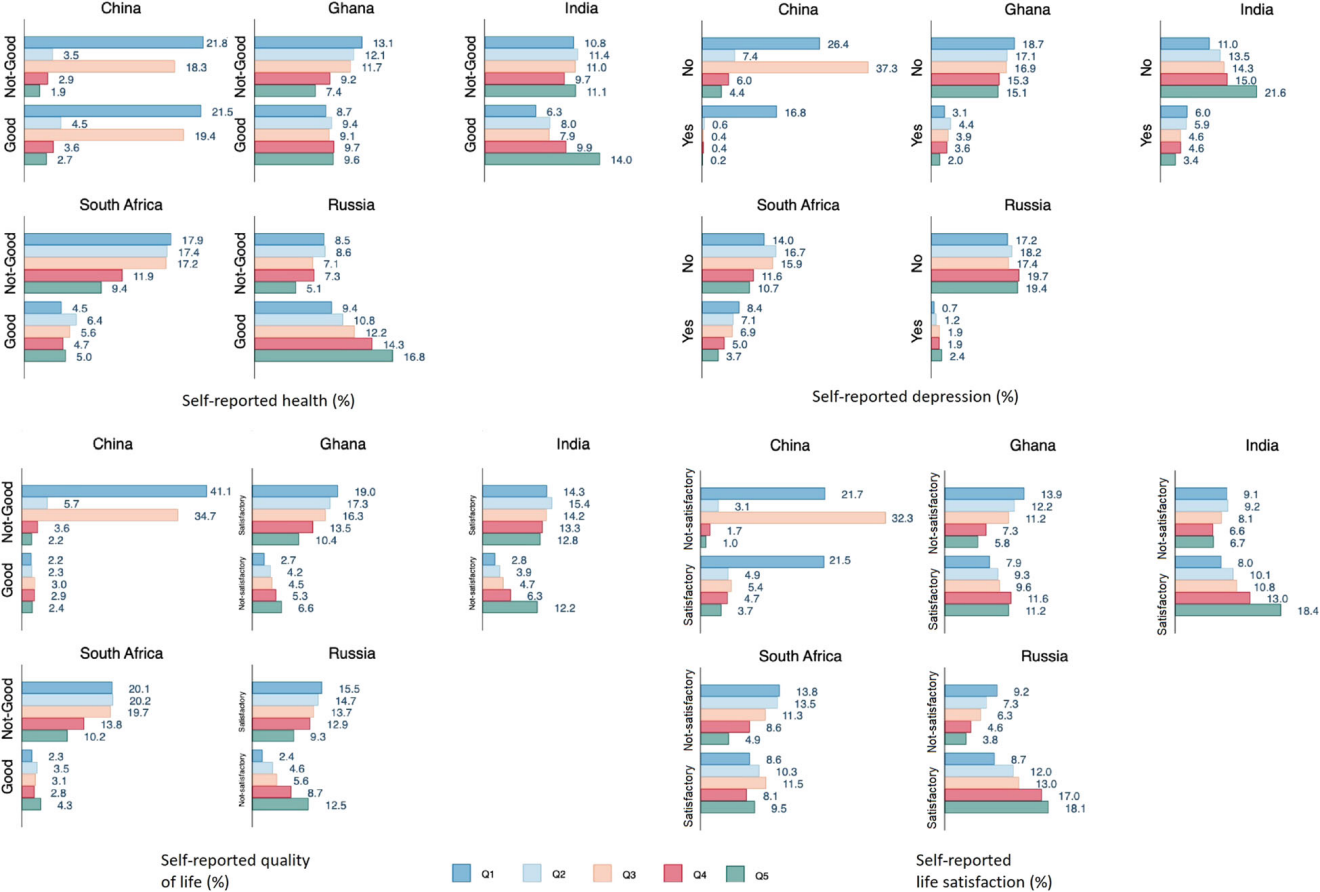
Prevalence of self-reported health, depression, quality of life and life satisfaction by income quintile (%)

Similar to subjectively measured financial stress, participants in the higher income quintiles were more likely to reported good health, quality of life, lower depression and satisfaction with life (Fig. 2)

Table 2 shows participants who reported varying degrees of financial difficulties in meeting daily needs e.g. moderate [Odds ratio = 0.81, 95% CI = 0.72,0.92], a little [Odds ratio = 0.39, 95% CI = 0.34,0.44], and not at all [Odds ratio = 0.25, 95% CI = 0.21,0.29], had significantly lower odds of good self-reported health (Model 1). Conversely, participants in the higher income quintiles (e.g. Q4, Q5) generally had higher odds of good self-reported health (Model 2). At country level analysis, the positive association was true for all countries but China. In model 3, the negative association between subjective financial stress and self-rated health was observed for all five countries; however, for income quintile, the positive association was observed only for Ghana [Odds ratio = 1.62, 95% CI = 1.13,2.32] and India [Odds ratio = 1.61, 95% CI = 1.14,2.27], while for China the association appeared to be negative [Odds ratio = 0.31, 0.20,0.48].

**Table 2:** Association between financial stress and good self-reported health

	Overall	China	Ghana	India	Russia	South Africa
Model 1						
**Has Money (Completely)**						
Moderately	0.81^***^ (0.72,0.92)	0.66^**^ (0.51,0.86)	0.47^**^ (0.28,0.77)	0.55^***^ (0.41,0.74)	0.48^***^ (0.37,0.63)	0.53 (0.28,1.01)
A Little	0.39^***^ (0.34,0.44)	0.13^***^ (0.09,0.17)	0.31^***^ (0.19,0.51)	0.29^***^ (0.21,0.40)	0.46^***^ (0.33,0.65)	0.22^***^ (0.12,0.41)
Not at All	0.25^***^ (0.21,0.29)	0.05^***^ (0.02,0.12)	0.22^***^ (0.13,0.36)	0.23^***^ (0.15,0.35)	0.24^***^ (0.16,0.37)	0.15^***^ (0.08,0.28)
Model 2						
**Income quintile (Q1)**						
Q2	0.81^**^ (0.71,0.93)	0.70^*^ (0.50,0.96)	1.27 (0.95,1.69)	1.29 (0.93,1.78)	1.38 (0.98,1.95)	1.08 (0.72,1.62)
Q3	0.98 (0.86,1.10)	0.53^***^ (0.40,0.70)	1.20 (0.89,1.60)	1.33 (0.96,1.86)	1.22 (0.86,1.74)	1.47 (0.96,2.25)
Q4	1.04 (0.90,1.19)	0.70 (0.49,1.01)	1.61^**^ (1.18,2.20)	2.06^***^ (1.48,2.88)	1.06 (0.72,1.56)	1.59^*^ (1.04,2.44)
Q5	1.46^***^ (1.26,1.70)	0.89 (0.59,1.33)	2.14^***^ (1.52,3.01)	2.00^***^ (1.40,2.85)	1.76^**^ (1.19,2.60)	2.38^***^ (1.48,3.82)
Model 3						
**Has Money (Completely)**						
Moderately	0.82^**^ (0.73,0.93)	0.61^***^ (0.47,0.80)	0.48^**^ (0.29,0.79)	0.56^***^ (0.42,0.76)	0.49^***^ (0.38,0.65)	0.56 (0.29,1.05)
A Little	0.39^***^ (0.35,0.44)	0.09^***^ (0.07,0.13)	0.33^***^ (0.20,0.53)	0.30^***^ (0.21,0.42)	0.48^***^ (0.34,0.68)	0.23^***^ (0.13,0.43)
Not at All	0.25^***^ (0.21,0.29)	0.03^***^ (0.01,0.08)	0.24^***^ (0.15,0.40)	0.25^***^ (0.16,0.39)	0.25^***^ (0.17,0.39)	0.16^***^ (0.08,0.30)
**Income quintile (Q1)**						
Q2	0.72^***^ (0.63,0.83)	0.41^***^ (0.29,0.58)	1.17 (0.87,1.57)	1.16 (0.83,1.61)	1.29 (0.90,1.83)	1.04 (0.68,1.59)
Q3	0.85^*^ (0.75,0.97)	0.41^***^ (0.30,0.55)	1.04 (0.77,1.41)	1.14 (0.81,1.61)	1.06 (0.74,1.53)	1.47 (0.95,2.27)
Q4	0.85^*^ (0.73,0.98)	0.27^***^ (0.18,0.40)	1.36 (0.99,1.88)	1.22 (0.83,1.79)	0.92 (0.62,1.37)	1.37 (0.88,2.14)
Q5	1.06 (0.91,1.24)	0.31^***^ (0.20,0.48)	1.62^**^ (1.13,2.32)	1.61^**^ (1.14,2.27)	1.48 (0.99,2.23)	1.55 (0.94,2.57)

Exponentiated coefficients; 95% confidence intervals in brackets.

^*^ *p* < 0.05, ^**^ *p* < 0.01, ^***^ *p* < 0.001

From Table 3 it is clear that adverse financial stress was strongly and positively associated with self-reported depression in all countries except for South Africa (Model 1). The odds of self-reported depression were higher for those who mentioned meeting daily financial needs ‘A little’ [Odds ratio = 1.97, 95% CI = 1.67,2.32], and even higher for those who mention ‘Not at all’ [Odds ratio = 2.66, 95% CI = 2.19,3.23]. On the other hand, those who were in the higher income quintiles (Model 2) had lower odds of self-reported depression, with the exception of South Africa where the odds were significantly higher for the highest income quintile [Odds ratio = 3.79, 95% CI = 1.61,8.89]. Similar associations were observed in Model 3, except the fact that higher income quintile increased the odds of self-reported depression for Ghana as well.

**Table 3:** Association between financial stress and self-reported depression

	Overall	China	Ghana	India	Russia	South Africa
Model 1						
**Has Money (Completely)**						
Moderately	1.03 (0.86,1.23)	0.41^**^ (0.24,0.70)	1.16 (0.54,2.45)	1.90^**^ (1.27,2.85)	1.31^*^ (1.01,1.70)	0.70 (0.33,1.47)
A Little	1.97^***^ (1.67,2.32)	3.07^***^ (1.95,4.86)	1.42 (0.70,2.90)	2.82^***^ (1.85,4.29)	1.74^***^ (1.29,2.34)	0.58 (0.28,1.19)
Not at All	2.66^***^ (2.19,3.23)	4.66^**^ (1.61,13.48)	2.96^**^ (1.45,6.05)	2.75^***^ (1.70,4.46)	2.98^***^ (2.19,4.06)	0.73 (0.33,1.58)
Model 2						
**Income quintile (Q1)**						
Q2	0.58^***^ (0.49,0.67)	0.23^***^ (0.14,0.39)	1.43 (0.97,2.10)	0.88 (0.63,1.22)	0.73^*^ (0.54,0.97)	1.25 (0.52,3.00)
Q3	0.26^***^ (0.22,0.31)	0.03^***^ (0.01,0.05)	1.28 (0.86,1.90)	0.61^**^ (0.43,0.87)	0.77 (0.57,1.03)	2.12 (0.93,4.85)
Q4	0.53^***^ (0.45,0.63)	0.20^***^ (0.11,0.36)	1.26 (0.83,1.93)	0.68^*^ (0.48,0.98)	0.92 (0.66,1.28)	2.07 (0.90,4.78)
Q5	0.33^***^ (0.27,0.40)	0.10^***^ (0.04,0.24)	0.58^*^ (0.35,0.96)	0.28^***^ (0.18,0.44)	0.65^*^ (0.45,0.94)	3.79^**^ (1.61,8.89)
Model 3						
**Has Money (Completely)**						
Moderately	1.11 (0.93,1.32)	0.68 (0.40,1.17)	1.11 (0.52,2.37)	1.61^*^ (1.06,2.44)	1.29 (,1.68)	0.79 (0.37,1.67)
A Little	1.92^***^ (1.62,2.27)	2.90^***^ (1.79,4.69)	1.34 (0.65,2.74)	2.19^***^ (1.41,3.40)	1.70^***^ (1.26,2.29)	0.78 (0.36,1.65)
Not at All	2.37^***^ (1.95,2.88)	2.30 (0.81,6.55)	2.82^**^ (1.36,5.82)	2.03^**^ (1.23,3.36)	2.87^***^ (2.10,3.93)	1.00 (0.45,2.26)
**Income quintile (Q1)**						
Q2	0.61^***^ (0.52,0.72)	0.29^***^ (0.17,0.49)	0.89 (0.52,1.51)	0.92 (0.66,1.29)	0.79 (0.59,1.07)	1.30 (0.52,3.24)
Q3	0.29^***^ (0.24,0.34)	0.03^***^ (0.01,0.06)	1.60^*^ (1.08,2.38)	0.65^*^ (0.45,0.93)	0.88 (0.65,1.20)	2.29 (0.97,5.44)
Q4	0.61^***^ (0.51,0.72)	0.30^***^ (0.16,0.57)	1.62^*^ (1.07,2.45)	0.77 (0.53,1.11)	1.02 (0.72,1.43)	2.35 (0.98,5.63)
Q5	0.40^***^ (0.33,0.49)	0.16^***^ (0.07,0.39)	1.67^*^ (1.08,2.59)	0.37^***^ (0.23,0.58)	0.78 (0.53,1.13)	4.09^**^ (1.65,10.13)

Exponentiated coefficients; 95% confidence intervals in brackets.

^*^ *p* < 0.05, ^**^ *p* < 0.01, ^***^ *p* < 0.001

The odds ratios of association between financial stress and good quality of life were presented in Table 4. The association between subjective financial stress and quality of life was consistently negative (Model 1), and consistently positive for higher income wealth quintiles (Model 2) for all five countries. In model 2, the strength of the association for income quintile (Q5) was noticeably high for China [Odds ratio = 10.21, 95% CI = 6.78,15.37]. However, after adjusting for subjective financial stress (model 3), the effect size was greatly diminished [Odds ratio = 3.94, 95% CI = 2.50,6.20].

**Table 4:** Association between financial stress and good quality of life

	Overall	China	Ghana	India	Russia	South Africa
Model 1						
**Has Money (Completely)**						
Moderately	0.26^***^ (0.23,0.30)	0.09^***^ (0.07,0.12)	0.30^***^ (0.19,0.48)	0.26^***^ (0.19,0.34)	0.24^***^ (0.17,0.34)	0.41^***^ (0.25,0.66)
A Little	0.14^***^ (0.12,0.16)	0.06^***^ (0.02,0.03)	0.17^***^ (0.11,0.26)	0.12^***^ (0.08,0.17)	0.24^***^ (0.16,0.37)	0.15^***^ (0.09,0.24)
Not at All	0.12^***^ (0.10,0.15)	0.09^***^ (0.004,0.17)	0.07^***^ (0.04,0.12)	0.10^***^ (0.06,0.16)	0.16^***^ (0.09,0.28)	0.14^***^ (0.09,0.23)
Model 2						
**Income quintile (Q1)**						
Q2	2.07^***^ (1.74,2.46)	3.05^***^ (2.16,4.31)	1.73^**^ (1.18,2.53)	1.19 (0.82,1.74)	1.48 (0.97,2.25)	1.84^*^ (1.10,3.06)
Q3	1.33^***^ (1.12,1.58)	1.08 (0.76,1.53)	1.88^**^ (1.28,2.77)	1.33 (0.90,1.95)	1.35 (0.88,2.09)	2.32^**^ (1.40,3.85)
Q4	3.65^***^ (3.08,4.34)	6.73^***^ (4.68,9.69)	2.53^***^ (1.71,3.75)	1.82^**^ (1.25,2.63)	1.49 (0.94,2.34)	4.14^***^ (2.52,6.79)
Q5	6.70^***^ (5.63,7.96)	10.21^***^ (6.78,15.37)	4.02^***^ (2.66,6.07)	3.19^***^ (2.19,4.67)	3.18^***^ (2.06,4.93)	6.46^***^ (3.84,10.86)
Model 3						
**Has Money (Completely)**						
Moderately	0.28^***^ (0.24,0.32)	0.10^***^ (0.08,0.14)	0.31^***^ (0.19,0.49)	0.28^***^ (0.21,0.37)	0.25^***^ (0.18,0.36)	0.42^***^ (0.26,0.69)
A Little	0.17^***^ (0.15,0.20)	0.04^***^ (0.02,0.06)	0.18^***^ (0.11,0.28)	0.13^***^ (0.09,0.19)	0.26^***^ (0.17,0.40)	0.18^***^ (0.11,0.29)
Not at All	0.15^***^ (0.12,0.18)	0.05^***^ (0.02,0.22)	0.09^***^ (0.05,0.14)	0.11^***^ (0.07,0.19)	0.18^***^ (0.10,0.31)	0.17^***^ (0.10,0.29)
**Income quintile (Q1)**						
Q2	1.86^***^ (1.55,2.23)	2.19^***^ (1.48,3.25)	1.50^*^ (1.01,2.23)	0.99 (0.67,1.47)	1.38 (0.89,2.13)	1.84^*^ (1.08,3.11)
Q3	1.33^**^ (1.11,1.59)	1.19 (0.81,1.74)	1.49 (1.00,2.22)	1.04 (0.70,1.56)	1.14 (0.72,1.78)	2.37^**^ (1.40,4.00)
Q4	3.07^***^ (2.57,3.68)	2.98^***^ (1.97,4.50)	1.88^**^ (1.25,2.85)	1.23 (0.83,1.82)	1.25 (0.78,2.00)	3.70^***^ (2.21,6.19)
Q5	5.09^***^ (4.24,6.11)	3.94^***^ (2.50,6.20)	2.51^***^ (1.62,3.90)	1.54^*^ (1.02,2.33)	2.57^***^ (1.63,4.07)	4.50^***^ (2.60,7.81)

Exponentiated coefficients; 95% confidence intervals in brackets.

^*^ *p* < 0.05, ^**^ *p* < 0.01, ^***^ *p* < 0.001

Regarding life satisfaction (Table 5), the association with subjective financial stress (Model 1) showed a consistently negative association both in the pooled and country-stratified analyses. Whereas for income quintile (Model 2), the associations were significantly positive for all countries, except for Q3 in overall [Odds ratio = 0.47, 95% CI = 0.41,0.53] and Chinese [Odds ratio = 0.12, 95% CI = 0.09,0.15] participants. In South Africa, those who were in the highest income quintile were more than five times [Odds ratio = 5.47, 95% CI = 3.32,9.01] as likely to report positive life satisfaction compared with those who were in the lowest. The association still remained noticeably strong [Odds ratio = 4.03, 95% CI = 2.38,6.81] even after adjusting for subjective financial stress (Model 3).

**Table 5:** Association between financial stress and positive life satisfaction

	Overall	China	Ghana	India	Russia	South Africa
Model 1						
**Has Money (Completely)**						
Moderately	0.25^***^ (0.22,0.29)	0.06^***^ (0.05,0.08)	0.61^*^ (0.37,0.99)	0.55^***^ (0.39,0.77)	0.39^***^ (0.31,0.50)	0.54 (0.27,1.07)
A Little	0.19^***^ (0.17,0.22)	0.03^***^ (0.02,0.06)	0.44^***^ (0.27,0.70)	0.26^***^ (0.18,0.37)	0.24^***^ (0.18,0.32)	0.22^***^ (0.11,0.42)
Not at All	0.14^***^ (0.12,0.16)	0.02^***^ (0.001,0.04)	0.24^***^ (0.15,0.39)	0.15^***^ (0.10,0.24)	0.15^***^ (0.11,0.21)	0.15^***^ (0.08,0.29)
Model 2						
**Income quintile (Q1)**						
Q2	1.00 (0.88,1.14)	0.95 (0.69,1.29)	1.42^*^ (1.06,1.88)	1.26 (0.92,1.73)	1.10 (0.83,1.46)	1.68^*^ (1.12,2.51)
Q3	0.47^***^ (0.41,0.53)	0.12^***^ (0.09,0.15)	1.51^**^ (1.13,2.03)	1.60^**^ (1.16,2.21)	1.45^*^ (1.09,1.93)	2.34^***^ (1.53,3.57)
Q4	1.81^***^ (1.57,2.08)	1.79^**^ (1.24,2.58)	2.79^***^ (2.04,3.83)	2.31^***^ (1.65,3.22)	1.09 (0.80,1.50)	4.02^***^ (2.56,6.29)
Q5	2.74^***^ (2.35,3.20)	2.72^***^ (1.73,4.26)	3.31^***^ (2.34,4.68)	2.86^***^ (1.99,4.10)	2.45^***^ (1.74,3.45)	5.47^***^ (3.32,9.01)
Model 3						
**Has Money (Completely)**						
Moderately	0.29^***^ (0.25,0.33)	0.09^***^ (0.07,0.13)	0.62 (0.37,1.01)	0.59^**^ (0.42,0.83)	0.40^***^ (0.32,0.51)	0.56 (0.28,1.14)
A Little	0.23^***^ (0.20,0.27)	0.05^***^ (0.04,0.07)	0.47^**^ (0.29,0.76)	0.29^***^ (0.20,0.42)	0.25^***^ (0.18,0.34)	0.27^***^ (0.14,0.53)
Not at All	0.15^***^ (0.13,0.18)	0.02^***^ (0.01,0.04)	0.30^***^ (0.18,0.49)	0.19^***^ (0.12,0.29)	0.16^***^ (0.11,0.22)	0.18^***^ (0.09,0.37)
**Income quintile (Q1)**						
Q2	0.91 (0.79,1.04)	0.64^*^ (0.45,0.91)	1.30 (0.98,1.74)	1.13 (0.82,1.56)	0.95 (0.70,1.28)	1.66^*^ (1.10,2.53)
Q3	0.44^***^ (0.38,0.49)	0.10^***^ (0.07,0.13)	1.31 (0.97,1.76)	1.36 (0.97,1.90)	1.19 (0.88,1.62)	2.24^***^ (1.45,3.47)
Q4	1.57^***^ (1.35,1.82)	0.71 (0.47,1.08)	2.33^***^ (1.69,3.22)	1.74^**^ (1.23,2.46)	0.90 (0.64,1.26)	3.57^***^ (2.24,5.67)
Q5	2.15^***^ (1.82,2.53)	0.92 (0.55,1.53)	2.51^***^ (1.75,3.61)	1.69^**^ (1.14,2.49)	1.98^***^ (1.37,2.85)	4.03^***^ (2.38,6.81)

Exponentiated coefficients; 95% confidence intervals in brackets.

^*^ *p* < 0.05, ^**^ *p* < 0.01, ^***^ *p* < 0.001

## Discussion

This study was dedicated to exploring how financial distress correlates with subjective health, depression, quality of life, and life satisfaction among older adults in China, Ghana, India, Russia and South Africa. We used data from the Study on Global Ageing and Adult Health (SAGE), which is a multi-country survey aiming to address the gap in reliable data and scientific knowledge on ageing and health in developing countries [18]. The sample population was limited to those aged 65 years and above. The initial descriptive findings indicate a noticeably low prevalence of good self-reported health, quality of life and positive life-satisfaction. This is explainable given the advanced age group and the high prevalence of non-communicable diseases. Almost three-quarter of the participants were living with at least one chronic condition. In addition to the health conditions, we observed that less than a fifth of the participants reported having enough money to meet daily their necessities completely, while only about 14% were in the highest income quintile. While interpreting these descriptive results, it is however important to bear in mind that the surveys were conducted in 2010, and therefore these prevalence rates have probably changed since then. With a few exceptions, the odds of reporting good self-reported health, quality of life, and life satisfaction were generally lower among those with varying degrees of financial stress, and larger among those in the higher income quintiles. Contrarily, the likelihood of self-reported depression was significantly higher among those with any level of financial stress, and lower among those in the higher income quintiles.

Both the descriptive and regression analyses revealed that the likelihood of reporting good health and quality of life were relative higher among those who reported meeting daily financial needs ‘Completely’ compared with those who mentioned having varying degrees of difficulty. The negative consequences of financial stress on physical and mental health are significant, especially among the older adults. Conversely, degeneration of physical and cognitive health due to aging also act as limiting factor of financial well-being. The challenge of promoting health and overall well-being of the older adults is beyond the scope of the scope of healthcare systems and requires policy attention such as old age pension, exemption from out-of-pocket medical expenditure and other social benefits to meet their special needs [41]. Our findings also indicated a positive correlation with financial stress and self-reported depression, and negative correlation with quality of life and life-satisfaction. It is important to bear in mind the strength of the associations varied considerably across the five countries, implying the role of local contextual factors that can mediate the relationship [42]. Therefore, more studies will be necessary to fully understand the pattern of the relationship between poverty and health, and well-being among older adults in low-resource countries.

Despite the well-documented association between financial stress and health outcomes, the literature on this topic is continuing to grow. Previous studies illustrate an inverse association between financial standing with subjective and objective measures of health [43–46] and quality of life [47–50] as well. However, a closer inspection reveals that the pattern of the association varies depending on the context and methodological approaches. This is most likely because poverty has a strong subjective component and estimating relative financial well-being is an inherently challenging task. One study by Oshio conducted on Japanese population reported that the conventionally definitions of poverty may underestimate the actual situation of poverty in terms of population health [46]. Importantly, the present study shows that both subjective and objective measures of financial stress are associated with health and quality of life, and provides interesting insights for further research by evaluating the relative sensitivity of poverty measures in predicting health and quality of life outcomes. Methodological heterogeneity is likely to remain a common concern for cross-cultural studies; as such, it is advisable that financial well-being should be evaluated from a multidimensional approach, capturing not only monetary conditions but also non-monetary conditions [45].

Increasing life expectancy, driven by improving socioeconomic conditions and living standards is triggering rapid population aging that is having repercussions on population health and healthcare systems. For low resource countries, adjusting policies to prioritize the health and financial needs of the elderly is an extremely challenging task, and failure to do so may translate to poor health and quality of life. Income poverty during advanced age is a common scenario, and its negative impact on health and well-being is widely recognised. The evidence generated by the present study furthers the current evidence base by providing a comparative situation between subjective financial insecurity as well as observed income inequality. The results should be interpreted with caution since the data were cross-sectional which precludes drawing any causal inference. This study therefore calls for more in-depth research to elaborate the mechanisms through which financial insecurity affects various aspects of physical, psychological and social well-being among this fast-expanding demographic sub-group in the developing countries.

An important aspect of this study is the use of subjective measures of the health and life related outcomes. All these constructs have been gaining increasing popularity in both clinical and social research for their ease of use and high sensitivity to clinical outcomes (morbidity and mortality). Measuring physical and mental health status, as well as quality of life in the context of clinical research is challenging due to factors such as interviewers time and skill, respondent comprehension, costs of measurement (collection of biomarkers) [51]. From this aspect, SRH provides a simple yet effective tool for assessing overall health status among general people. In contrast, there are psychometrically validated constructs for measuring health-related quality of life (e.g. PROMIS GH [52], International Quality of Life Assessment [53], SF-36 [38]). PROMIS GH contains 10 items covering Global Physical Health (GPH) and Global Mental Health (GMH) and were found to be sensitive enough to detect longitudinal changes in health form clinical conditions including chronic diseases [54, 55].

This study has some important strengths and limitations to report. We had comparable data from five low-middle income countries (LMICs), which increases the scope of the study and provides a better contrasting pictures of the association between financial stress with health and quality of life. Large scale population-based studies are hard to conduct across multiple countries. World Health Organization’s open data policy is easing data constraints among researchers in LMICs and helping to better understand the influence of socioeconomic factors on population health outcomes. Among the limitations were age and secondary nature of the surveys. Data were secondary and therefore the authors had no control over the selection and measurement of the study variables. As such, factors such as cultural, dietary, and environmental variables were not adjusted for which are strongly correlated linked with health and quality of life. The variables were measured based on participants own assessments, and hence remain subject to recall bias and overreporting/underreporting. Finally, the surveys were cross-sectional and hence the associations may not any indicate any causal relationship.

## Conclusion

Using data from a multi-country survey from Study on Global Ageing and Adult Health (SAGE), this study assessed the relationship between financial stress and subjective health, depression, quality of life, and life satisfaction among older adults in five developing countries. Except for a few inconsistencies, we found that both subjectively and objectively measured financial insecurity correlate negatively with good self-reported health, quality of life, life satisfaction, and positively with self-reported depression. The strength of the associations varied considerably by the levels of financial stress, and across countries. Although the analysis cannot guarantee any causality of the findings, it is recommendable that the issue of material poverty among the older adults is given special attention especially in the fast-developing countries which are experiencing rising life expectancy and healthcare and social challenges associated with aging.

## Data Availability

Data are available through the WHO SAGE website.
